# Density of Population

**Published:** 1912-05

**Authors:** 


### Density of Population

IN a previous editorial, dealing with openings for medical men in the country, we made a rough estimate of 30 per square mile for rural population. The Bureau of the Census has just issued an interesting Bulletin regarding the population of New York State. The actual rural population proves to be as follows: (ignoring fractions.

| | | |
|---|---|---|
| Erie Co. .......69 | Monroe Co. ....88 | Livingston Co. ...50 |
| Niagara Co. ...60 | Genesee Co. ...45 | Ontario Co. .....50 |
| Chautauqua Co. 43 | Orleans Co. ...54 | Wyoming Co. ....40 |
| Cattaraugus Co.34 | Wayne Co. ....61 | Seneca Co. .......49 |

But as the inhabitants of villages of less than 2,500 are included in the rural population, our estimate is probably nearly correct.

*Actually the death rate of Albany is much higher than the average.

The Bulletin, however, deserves study from a broader sociologic standpoint. Ignoring, for present purposes, the matter of urban population, which also has a paramount influence on the total density of population for any county containing a good-sized city, we note the following points of interest regarding rural (including village) population:

The most sparsely populated county is Hamilton: 2.6 per sq. mile. The adjacent counties: Lewis, Hamilton, Herkimer and Essex, range up to 18 per square mile.

The seven counties in or near New York City either have no truly rural population at all (in the inclusive sense defined) or have, even outside of towns of over 2,500, a population at least as dense as 90 per square mile.

Otherwise, the rural population of the whole state ranges from 18 to 90 per square mile, the counties of the southern tier and the northern curve ranging below 45, most of the central and western counties above 45.

In regard to proportionate increase of population, it is noteworthy that New York City includes a trifle more than 52 per cent. of the entire population, having risen from 39 per cent. in 1890. In the same period, the distinctly urban population from the sociologic standpoint, i. e., in cities of 100,000 and more, aside from New York, has increased from 6.5 per cent. to 9.6 per cent. In cities of 10,000 up to 100,000, there has been a slight reduction, from 13.2 to 12.3 per cent. In smaller towns, there has been a moderate increase in number, especially of those between 2,500 and 5,000, but a slight decrease of percentage of aggregate population. Of the rural population including that of villages of less than 2,500, there has been an actual diminution of about 250,000 in 20 years, and a percentage decrease from 30 to 17 per cent.

Fifteen counties show an actual decrease in total population in the last ten years, in some insignificant, in others amounting to more than 10 per cent. But about 35 counties show a decrease in rural population.

We must confess, however, not to feel the apprehension as to the future welfare of the country, which such statistics arouse in the orthodox. To a considerable degree, the concentration of population in cities of the second magnitude, by which term we mean, approximately 100,000 to 500,000, is due to the normal growth of individual communities. One city swinging from 90,-000 to 100,000, as it properly should, in a decade, increases the distinctly urban population by considerably more than 1 per cent. and correspondingly diminishes the population of the next group, taking the total population of the State as 100 per cent. There are now four cities between 50,000 and 100,000, with an average population of over 75,000. A perfectly wholesome industrial growth of these would throw 400,000 more of our population from one arbitrary division to another by the next census and, if we estimate the total population of the State at about 11,000,000 in 1920, this will mean an addition of about 4 per cent. to the higher division and a corresponding subtraction from the lower.

Towns from 10,000 down, on the whole, show a tendency to diminution. To some extent, this is relative, due to the greater drawing power of large cities; to some extent it is due to the passing of a prosperous town from one division to a higher one. In some cases, there is an actual lessening of population. For such sociologists as deplore this tendency, we would recommend a sojourn of some months in the average town of a few thousand. We think they would then realize that if a town of this size does not tend by its own momentum to pass into a higher division, it is no place for an ambitious young man or woman or family. Indeed, the village is neither hay nor grass. Unless transformed into a miniature city by some industrial enterprise, it does not directly produce anything and unless controlled by an unusually powerful and successful public spirit, it possesses most of the temptations of a city and few of the uplifting influences of the latter.

As to the decline of the rural population, it should be remembered that the open country is, virtually, a factory of hay, grain, vegetables, fruits, wool and meat. Why should it be overmanned? Why should not the invention of labor saving machinery result in an economy of labor here as well as in a factory producing steel, automobiles, clothing, typewriters, etc? Instead of deploring, in glittering generalities, the evil of concentration of population in cities, why not look at the matter from the common-sense standpoint of utilizing the energy of its inhabitants and of enabling them to do the best for themselves and for the community at large. We may be pardoned a personal application. Yates Co. shows a decrease of over 10 per cent. of its population in the last ten years. Except Penn Yan, there is not a community in it that would give full scope to the average man, except for a few physicians, lawyers, clergymen, teachers, and the ordinary personnel of a small village. There is no water power or other natural advantage that would warrant the development of more than minor commercial enterprises, such as the manufacture of grape baskets and the usual run of local businesses for the convenience of an agricultural population. A good deal of what was once farming land on side hills, has been converted into vineyards, resulting in the great advantage of the owners. Labor in vineyards is largely done by non-residents, in the late summer season, redounding to the health of the laborers and not materially interfering with their regular and more profitable, labor in factories, offices and even schools—for many are students and teachers—in cities. We are familiar with every foot of a farm which, some sixty years ago, supported six related families. It is now conducted by one family, more productively, and supports about six persons. Probably the six families multiplied by two or three generations of increase, could still find the necessities of life on the same farm but they would be living on a scale little better than that of the Chinese peasantry. As it is, the same families represent the conduct of one of the most influential newspapers in the land, one of the most practical minor developments of electricity, educational work of the highest grade in several institutions, professional and commercial as well as agricultural industry in several states. Let sociology dry its tears on the handkerchief of common sense.

There is one more point, of special interest to the medical profession. The death rate—which means also the rate of incidence of disease and, in a negative sense, human efficiency and happiness—is now slightly less for the urban than for the rural population. When we recall the tendency of the superannuated farmer to withdraw to a town to spend the last few years of life; and count the number of the fatal cases charged to cities, but which have come from the country to make a last desperate effort for cure by operation or expert medical treatment, we must conclude that the strictly urban mortality is really a good deal less than that of the open country. The trite moralizing of the fourth reader must be revised.

